# Piezoelectrically Enhanced Photocatalysis with BiFeO_3_ Nanostructures for Efficient Water Remediation

**DOI:** 10.1016/j.isci.2018.06.003

**Published:** 2018-06-08

**Authors:** Fajer Mushtaq, Xiangzhong Chen, Marcus Hoop, Harun Torlakcik, Eva Pellicer, Jordi Sort, Chiara Gattinoni, Bradley J. Nelson, Salvador Pané

**Affiliations:** 1 Multi-Scale Robotics Lab (MSRL), Institute of Robotics and Intelligent Systems (IRIS), ETH Zurich, CH-8092 Zurich, Switzerland; 2 Departament de Física, Facultat de Ciències, Universitat Autònoma de Barcelona, Campus UAB, E-08193 Bellaterra (Cerdanyola del Vallès), Spain; 3 Institució Catalana de Recerca i Estudis Avançats (ICREA), Passeig Lluís Companys 23, E-08010 Barcelona, Spain; 4 Departament de Física, Universitat Autònoma de Barcelona, E-08193 Bellaterra (Cerdanyola del Vallès), Spain; 5 Materials Theory, ETH Zurich, CH-8093 Zurich, Switzerland

**Keywords:** Chemistry, Catalysis, Environmental Nanotechnology

## Abstract

Designing new catalysts that can efficiently utilize multiple energy sources can contribute to solving the current challenges of environmental remediation and increasing energy demands. In this work, we fabricated single-crystalline BiFeO_3_ (BFO) nanosheets and nanowires that can successfully harness visible light and mechanical vibrations and utilize them for degradation of organic pollutants. Under visible light both BFO nanostructures displayed a relatively slow reaction rate. However, under piezocatalysis both nanosheets and nanowires exhibited higher reaction rates in comparison with photocatalytic degradation. When both solar light and mechanical vibrations were used simultaneously, the reaction rates were elevated even further, with the BFO nanowires degrading 97% of RhB dye within 1 hr (*k*-value 0.058 min^−1^). The enhanced degradation under mechanical vibrations can be attributed to the promotion of charge separation caused by the internal piezoelectric field of BFO. BFO nanowires also exhibited good reusability and versatility toward degrading four different organic pollutants.

## Highlights

• Use of photocatalytic and ferroelectric BiFeO_3_ nanosheets and nanowires
• Harnessing solar energy and mechanical vibrations for organic pollutant removal
• Elevated performance under mechanical stress was attributed to the piezotronic effect
• Hydroxyl radicals and holes were dominant species for degradation of organics

## Graphical Abstract

**Figure:**
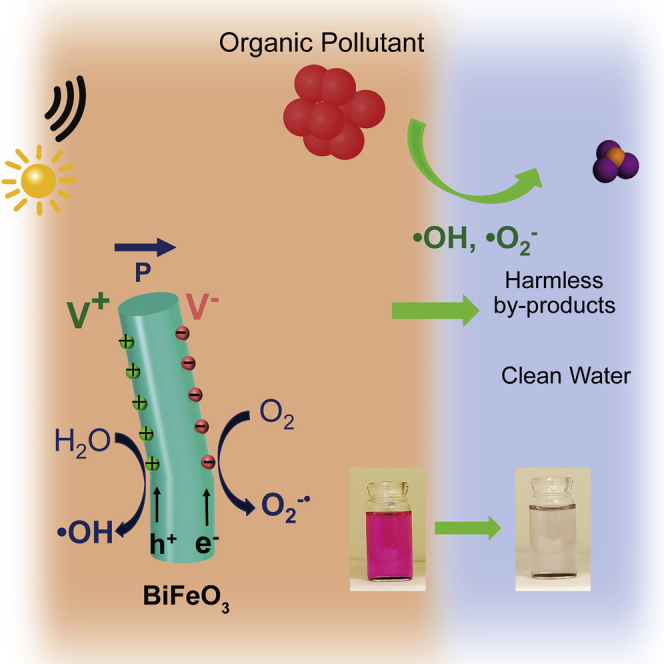

## Introduction

Environmental pollution and shortage of clean energy are among the most pressing problems that threaten sustainable development of human civilization. Water pollution caused by discharge of toxic, synthetic dyes into effluents is one of the major sources of environmental pollution (Matilainen et al., 2010). The presence of even trace amounts of these synthetic dyes in water is extremely harmful because of their carcinogenic and mutagenic nature (de Lima et al., 2007, Umbuzeiro et al., 2005, Ekici et al., 2001, Chung, 1983). Owing to their high solubility and chemical stability, most of these synthetic dyes easily escape conventional water treatment methods and persist in the environment (Fracasso et al., 1992). Advanced oxidation process (AOP) is a cost-effective and green approach to degrade such toxic dyes into harmless products, such as CO_2_ and H_2_O, using highly reactive species, including hydroxyl and superoxide radicals (Julkapli et al., 2014, Dong et al., 2007).

Photocatalysis is one of the most extensively researched fields of AOP, in which a semiconductor with a suitable bandgap can efficiently absorb light and form photogenerated electron-hole pairs. These electrons and holes can then migrate to the surface of the photocatalyst and initiate the oxidative/reductive processes, resulting in degradation of organic pollutants (Yan et al., 2013). One of the main drawbacks that limits practical use of photocatalysts is the high electron-hole recombination rate, which ultimately lowers their photocatalytic efficiency. To overcome this problem, wide bandgap semiconductors, such as titanium dioxide (TiO_2_), are extensively used. However, having a wide bandgap limits the light absorption of these materials to the UV region (Núñez et al., 2017). Hence, researchers have proposed new strategies, including developing novel nanostructures, using co-catalysts (Pt, Pd, and RuO_2_), doping with rare-earth or transition metals, and fabricating heterojunctions to tune the bandgap of these materials, to lower the electron-hole recombination rate, and to increase the lifetime of the charge carriers (Long and Prezhdo, 2015, Sakthivel et al., 2004, Mushtaq et al., 2015, Mushtaq et al., 2016, Zhang et al., 2011). Although such approaches can improve the separation of photogenerated electrons and holes, they rely heavily on the use of expensive catalysts such as noble metals and suffer from complicated fabrication processes, which, in turn, create obstacles to their practical application (Wang et al., 2016).

Apart from chemically modifying the wide bandgap, physical methods such as the application of external electric fields to an electrochemical cell were also employed to separate the electron-hole pairs and, hence, improve their photocatalytic performance (Barroso et al., 2013, Pesci et al., 2013). Even though this method has demonstrated strong potential to increase the photocatalytic efficiency, its high cost, complicated device structure, and difficult operation conditions provide significant challenges for practical use (Li et al., 2014, Hong et al., 2016, Wang et al., 2016).

Generating a localized electric field directly on a photocatalyst's surface is a more practical approach than applying a macroscale electric field in a chemical cell because of cost and lower energy consumption. Combining piezoelectric materials with visible light photocatalysts is one way to achieve this. Piezoelectric materials can generate an internal electric field under strain and, hence, induce separation of photogenerated electric charges (Xue et al., 2015, Hong et al., 2010, Singh and Khare, 2017, Yun et al., 2018, Nan et al., 2017). This approach was used to fabricate primarily core-shell nanostructures, in which the core was composed of piezoelectric materials, such as ZnO, BaTiO_3_, and NaNbO_3_, whereas the shell consisted of visible light photocatalysts, including CuS, FeS, and AgO_2_. In this approach, it was possible to achieve enhanced reaction efficiency by using the dual stimuli of light and mechanical vibrations (Hong et al., 2016, Xiao et al., 2016, Li et al., 2015, Jia et al., 2018). However, fabrication of such dual-phase core-shell nanomaterials raises further complications, such as complex synthesis and weak mechanical coupling between the piezoelectric and photocatalytic counterparts under strain for extended periods of time.

Low bandgap (i.e., visible light photocatalytic properties) and piezoelectric properties can co-exist in a single material. The use of BiFeO_3_ (BFO) as a promising candidate for visible light photocatalysis has been demonstrated owing to its low bandgap of ∼2.1 eV (Xian et al., 2011, Soltani and Entezari, 2013, Mocherla et al., 2013). In addition, BFO also possesses good piezo/ferroelectric performance with a large spontaneous polarization in excess of 100 μC cm^−2^ and piezoelectric coefficient (*d*_*33*_) of about 100 pm/V (Singh et al., 2006, Jung Min et al., 2012). We assume these properties render BFO an excellent candidate for efficiently using both light and vibrational energy for catalytic degradation of organic pollutants, without the need for coupling it to other materials or using an external bias (Qi et al., 2018).

In this work, we fabricated pure, single-crystalline BFO nanosheets (NSs) and nanowires (NWs), which are both visible light photoactive and piezoelectric. These BFO nanostructures were able to harness photonic as well as mechanical energy for the degradation of model organic pollutants such as rhodamine B (RhB). By coupling their photocatalytic and piezoelectric properties, degradation of RhB dye was greatly enhanced. Reactive species trapping experiments revealed the underlying mechanism of their catalytic performance. Development of these nanostructures contributes to the use of green technologies, such as harnessing natural sunlight and scavenging waste energies, such as noise and vibrations, for efficient environmental applications.

## Results and Discussion

**Figure 1:**
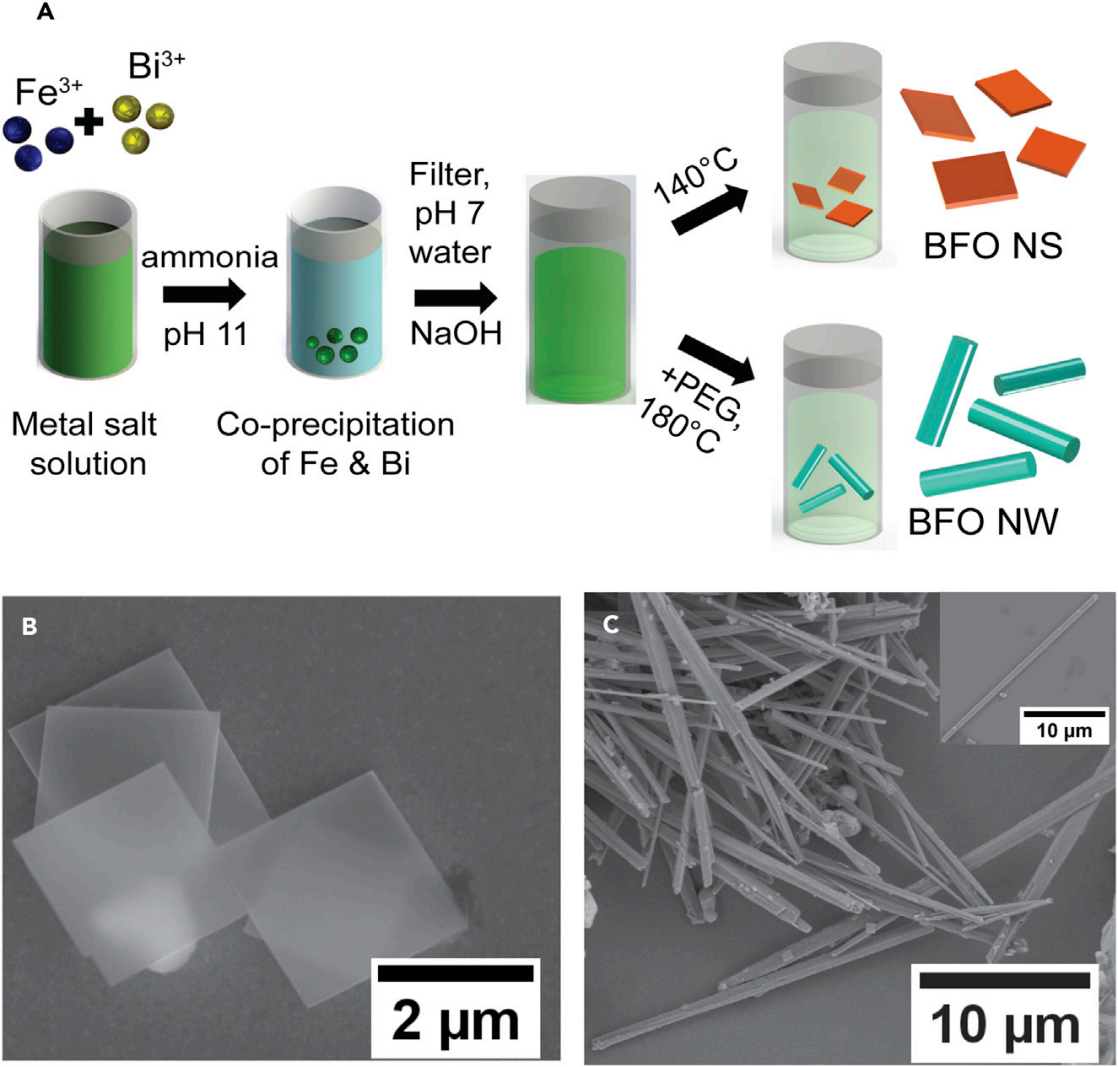
Fabrication Scheme and Morphology of BFO NSs and NWs (A) Scheme showing how to fabricate BFO NSs and NWs using hydrothermal synthesis. (B) Scanning electron microscope image showing overlapped BFO NSs. (C) Scanning electron microscope image showing several BFO NWs. In the inset a single NW is shown.

BFO NSs and NWs were fabricated by a hydrothermal synthesis approach by carefully tuning growth conditions, including surface chemistry, temperature, and duration of the reaction. The fabrication scheme of BFO NSs and NWs is presented in Figure 1A. First, concentrated ammonia was added to a solution of bismuth and iron salts to achieve co-precipitation of Bi^3+^ and Fe^3+^ ions (Li et al., 2012, Sun et al., 2014). Then a mineralizer (5 M NaOH) was added to the solution. The mixture was then sealed in an autoclave and placed in an oven at elevated temperature for hydrothermal treatment. Hydrothermal synthesis uses the dissolution-crystallization mechanism. Under hydrothermal conditions, the precipitates reacted with NaOH to dissolve and form complex ions. This was followed by the formation of BFO crystals in the supersaturated region by nucleation, precipitation, dehydration, and growth of these complex ions over time (Xu et al., 2016). For BFO NSs, the reaction temperature was 140°C and the reaction time was 72 hr. BFO NWs were fabricated by a surfactant-assisted hydrothermal route at 180°C for 72 hr. In this approach, the soft-templating effect of polyethylene glycol (PEG) was employed to direct the growth of BFO crystals to form BFO NWs (Harraz, 2008, Song et al., 2014). BFO NS fabricated in this study are rhombic with an edge length of 2–3 μm and a thickness of about 150 nm (Figure 1B). The BFO NWs are about 30 μm long, with an average diameter between 200 and 700 nm (Figure 1C).

**Figure 2:**
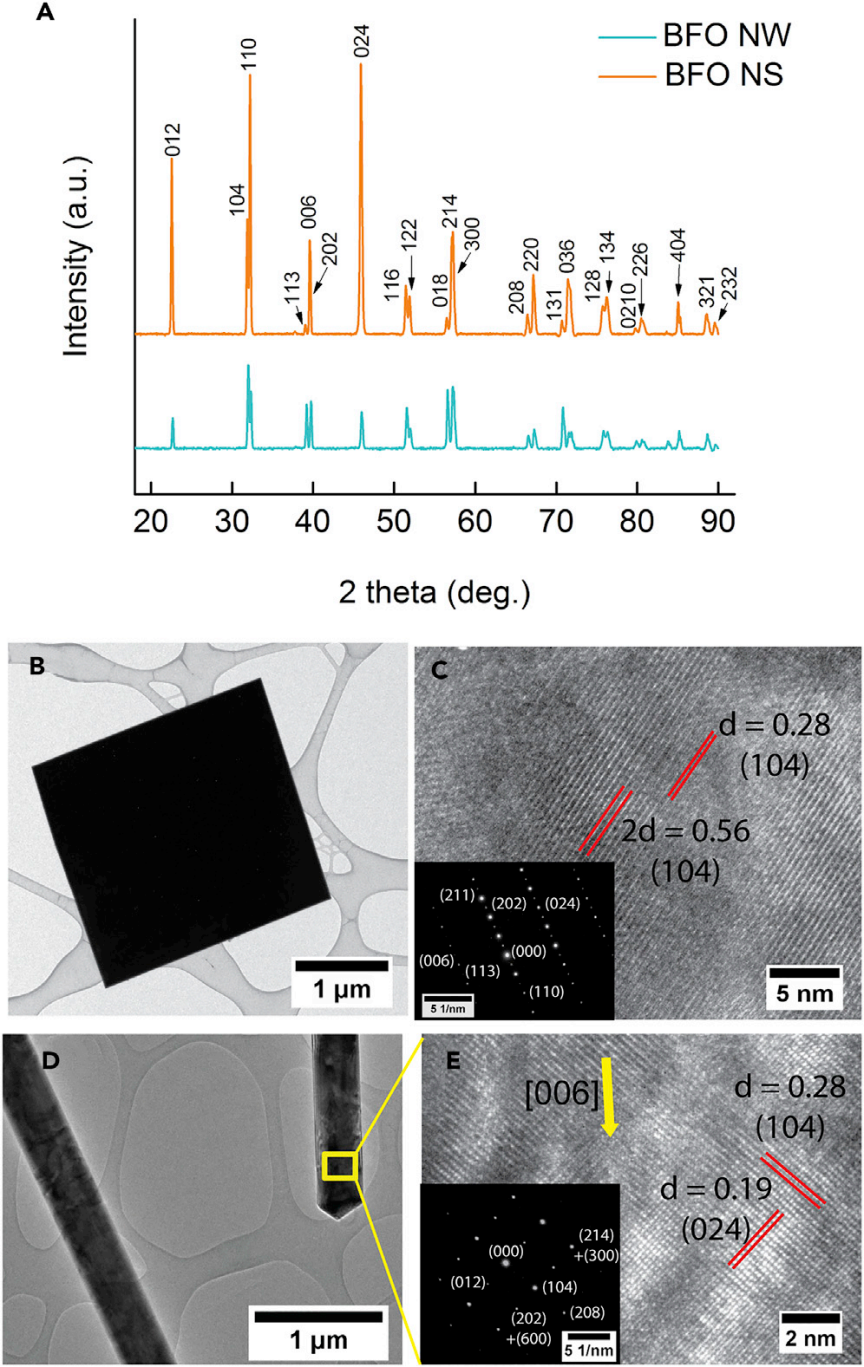
Structural Characterization of BFO NS and NW Samples (A) XRD patterns obtained for BFO NSs and NWs. (B) TEM image showing a single BFO NS. (C) The corresponding HRTEM image. Its electron diffraction pattern is shown in the inset. (D) TEM image showing two BFO NWs. (E) HRTEM image obtained from the marked area in Figure 2D, showing the growth direction of the NW along the [006] direction. The inset shows its electron diffraction pattern.

The crystalline structure of the BFO NSs and NWs were analyzed using X-ray diffraction (XRD) and transmission electron microscopy (TEM). XRD investigation performed on BFO nanostructures (Figure 2A) showed that for both NS and NW structures all peaks can be assigned to the pure phase of BiFeO_3_ (JCPDS No. 82–1254), indicating a rhombohedral perovskite structure with the space group *R3c.* Further evaluation of the BFO NS sample shows that the NSs have an average coherent diffraction length of 137 ± 10 nm and cell parameters *a* = *b* = 5.58567 ± 0.00002 Å and *c* = 13.88767 ± 0.00005 Å. A similar analysis performed on a BFO NW sample shows an average coherent diffraction length of 117 ± 10 nm and cell parameters *a* = *b* = 5.58599 ± 0.00003 Å and *c* = 13.88635 ± 0.00004 Å. TEM analysis of the BFO NS sample shows the presence of well-defined NS (Figure 2B), in agreement with scanning electron microscopy observations. A high-resolution TEM (HRTEM) image of a single NS is presented in Figure 2C and features an intact and orderly structure. The inset is the corresponding selected area electron diffraction (SAED) pattern, which confirms the single-crystalline structure. The spots in the SAED pattern have been indexed according to the *R3c* structure, and the result is compatible with a [024] growth direction of the NS. This is in agreement with the observed texture in the XRD pattern (Figure 2A) and in line with other reports from the literature on BFO NSs (Yang et al., 2014). The TEM image obtained for the BFO NW sample shows that the as-prepared nanopowder consists of straight NWs with a tip-like ending, suggesting a [006] growth direction (Figure 2D). Its corresponding HRTEM image corroborates the presence of an intact, orderly, single-crystalline structure. The planes with interplanar d-spacings of 0.28 nm and 0.19 nm match the (104) and (024) crystal faces, respectively (Figure 2E). The inset presents the corresponding SAED pattern, showing the single crystalline nature of BFO NW and further confirming the growth along the [006] direction (also see Figure S2).

**Figure 3:**
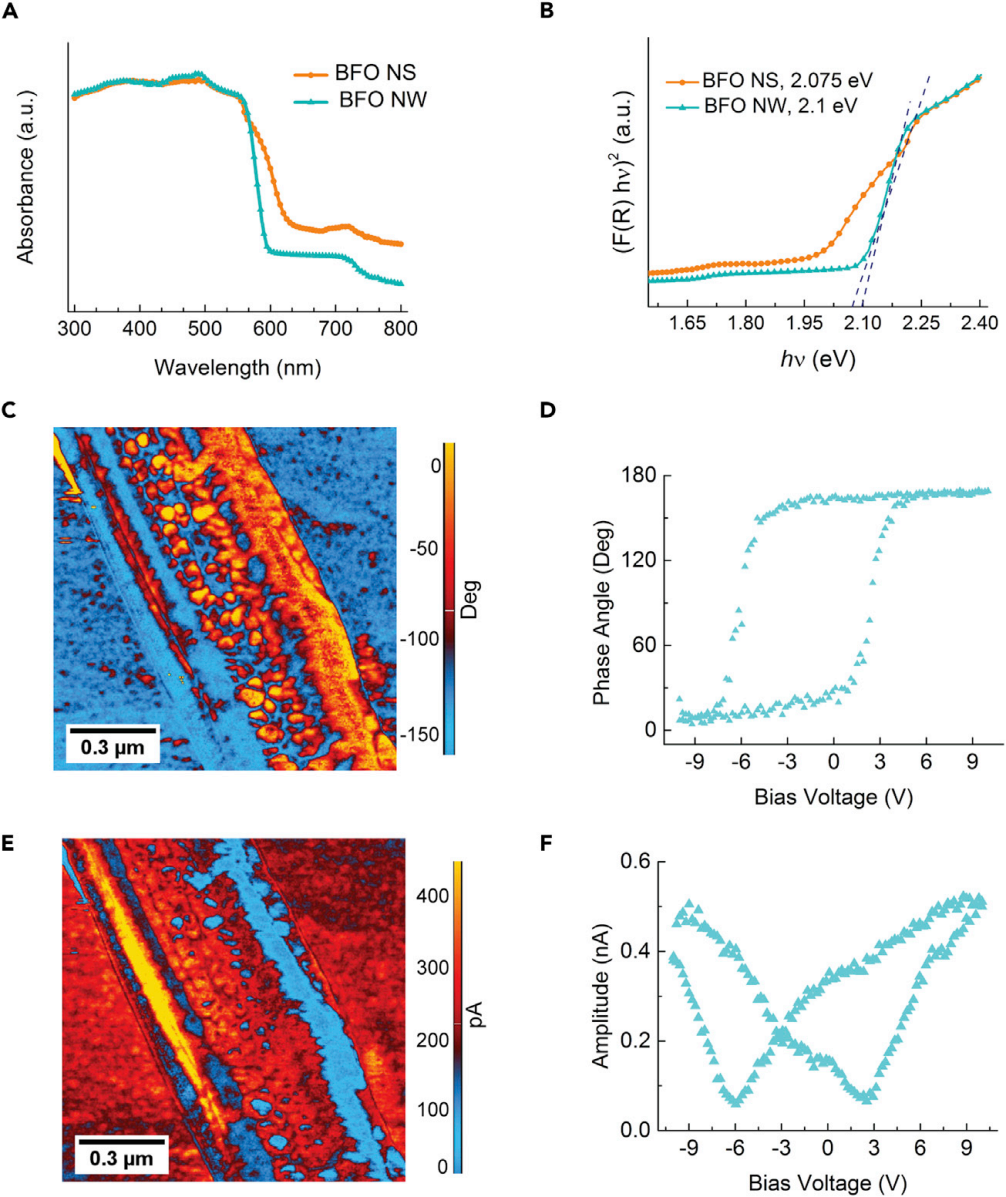
Optical and Piezoelectric Characterization of BFO Nanostructures (A) UV-Vis DRS spectra obtained for BFO NS and NW samples. (B) The corresponding Kubelka-Munc plot. (C and D) PFM characterization of BFO NW showing the phase response. (E and F) PFM characterization of BFO NW showing the amplitude response.

The light absorption properties of BFO NS and NW samples were investigated using UV-visible diffuse reflectance spectra (DRS) at room temperature. From Figure 3A, we can observe that both BFO nanostructures exhibit strong absorption in the UV and visible light region. Careful observation reveals that in comparison with the BFO NWs, the BFO NSs display a higher absorbance in the visible light region. The bandgaps of BFO NSs and NWs were calculated using the Kubelka-Munc function ([αhʋ]^2^ vs photon energy [hʋ]) for the direct bandgap semiconductor (Figure 3B). The bandgaps were estimated to be 2.075 eV for BFO NSs and 2.1 eV for BFO NWs, which are consistent with the literature (Soltani and Entezari, 2013, Xian et al., 2011). These small bandgap values for BFO NSs and NWs indicate that they can be used as photocatalysts under visible light.

The piezoelectricity of a single BFO NW was directly probed using piezoresponse force microscopy (PFM). A conductive cantilever tip was used in contact mode to apply an alternating voltage to the sample and induce piezoelectric surface oscillations. These oscillations were sensed through the consequent cantilever deflection. Figure 3 presents the PFM phase (C) and amplitude (E) images for BFO NWs. From the PFM phase images, a clear phase contrast between different regions of the NW can be observed. These regions with different contrast represent the domains with opposite out-of-plane polarization orientations. The amplitude image (Figure 3E) also shows the presence of distinct and randomly distributed ferroelectric domains. To further investigate the ferroelectric nature of our BFO samples, local piezoresponse hysteresis loops were obtained by sweeping the applied DC bias while simultaneously measuring the phase and amplitude response. The excitation voltage waveform was a stepwise increasing pulsed DC voltage, superimposed on a small AC voltage. To minimize the possible interference caused by electrostatic forces, the AC response signal was acquired only during the off-phase of the voltage pulse sequence (Chen et al., 2013). From the phase loop presented in Figure 3D it can be clearly observed that the polarization can be switched to the opposite direction by sweeping the tip bias. The average phase contrast is close to 180°, confirming that the measured signal originates from the electromechanical response rather than from electrostatic forces (Chen et al., 2017, Avinash et al., 2011). The amplitude of the response signal in PFM is directly related to the local strain of the BFO nanostructures (Figure 3F). This amplitude versus bias voltage curve is also hysteretic, and its shape resembles a butterfly loop, which is a well-established characteristic of ferroelectric materials (Xie et al., 2008). The piezoresponse phase and amplitude loops for BFO NWs are horizontally shifted. This asymmetry in the loops can be attributed to many factors, including the imprint effect, internal bias fields inside the materials, and/or a work function difference between the top Pt-coated Si probe and the bottom gold electrode (Chen et al., 2016). The well-defined piezoresponse hysteresis loops confirm the ferroelectric nature of our BFO NW samples. For NSs, similar analysis was performed and is presented in Figure S3.

**Figure 4:**
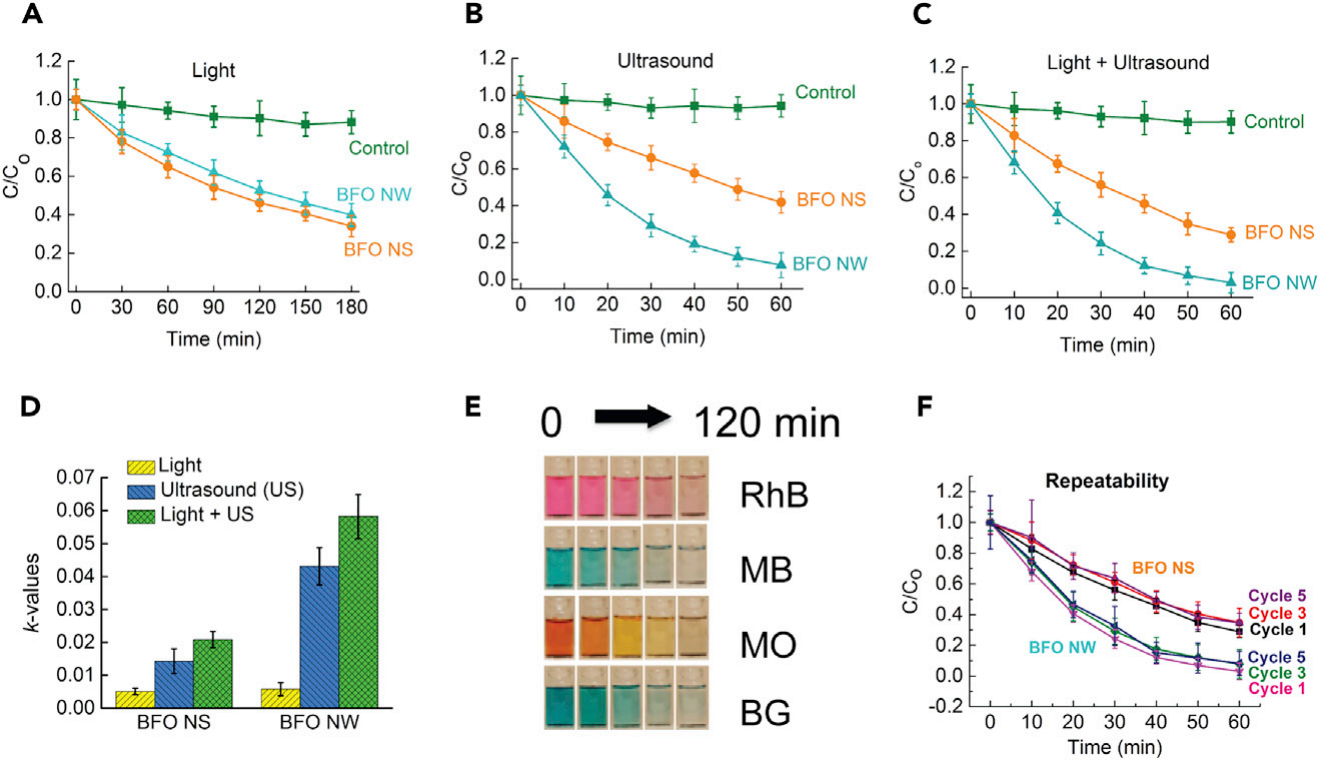
Photo and Piezocatalytic Degradation of RhB Dye Using BFO NSs and NWs (A) Catalytic degradation curve of RhB using light (n = 5). (B) Catalytic degradation curve of RhB using ultrasound (n = 5). (C) Catalytic degradation of RhB using a combination of light and ultrasound stimuli (n = 5). (D) Comparison of degradation rate constants of RhB obtained for BFO NS and NW under light, ultrasound, and their combination (n = 5). (E) Visual color change of four different organic dyes in 2 hr under dual stimuli using BFO NWs. (F) Five consecutive degradation curves obtained for BFO NS and NW under dual stimuli showing a good reusability trend (n = 5).

The ability of BFO nanostructures to degrade RhB using light and mechanical vibrations was investigated. Both BFO samples gradually degrade RhB dye under UV-visible light over 3 hr (66% for NS and 60% for NW samples). In contrast, the control sample without BFO displayed a negligible response (Figure 4A). The photocatalytic degradation rate obtained for BFO NS is slightly higher, which can be explained by its lower bandgap value of 2.075 eV in comparison with the NW sample (2.1 eV). Figure 4B presents the piezocatalytic response of BFO samples under ultrasonic wave vibrations. Both BFO samples exhibit a higher RhB degradation rate in comparison with photocatalytic degradation, whereby a 59% and 92% degradation was observed for BFO NS and NW samples in 1 hr, respectively. When the stimuli of light and mechanical vibrations are used simultaneously, the degradation efficiency of the organic dye solution can be further increased to 71% for NSs and 97% for NWs (Figure 4C). A quantitative analysis of these degradation rates is given by comparing their reaction rate constant *k*, which can be defined by

(Equation 1) $$k=\ln\frac{Co}{C}/t\;$$

where *C*_*o*_ is the initial RhB concentration and *C* is the RhB concentration at time *t*. This calculation is based on the assumption that the kinetics of the RhB degradation reaction catalyzed by the BFO nanostructures are (pseudo)-first-order reactions (Mushtaq et al., 2015, Mushtaq et al., 2016). These results are summarized in Figure 4D, where we can observe that both BFO NS and NW samples display similar photocatalytic rate constants (0.0058 and 0.0051 min^−1^, respectively). However, under ultrasonic stimulation two interesting phenomena can be observed. First, the RhB degradation rates obtained for both BFO nanostructures are much higher under the piezocatalytic effect than under the photocatalytic effect (2.5 times higher for BFO NS and 8.5 times for BFO NW). Such an enhancement can be explained by the piezotronic effect, which modulates the electrical transporting property at the BFO-electrolyte heterojunction using BFO's internal electric bias. This acts as a driving force for separating the electrons and holes generated by piezocatalysis and, thus, enhances the performance of photochemical reactivity of BFO (Wang et al., 2016, Xue et al., 2015). These surface charges are locally confined, allowing for the chemical reactions to degrade the dye molecules. A second interesting result is that the *k*-value of BFO NWs (0.0431 min^−1^) is three times higher than that of the BFO NSs (0.0143 min^−1^). We assume that, under mechanical stress the high-aspect-ratio BFO NWs experience a greater strain and, hence, a higher strain-induced voltage in comparison with the NS sample. This assumption was verified by using the COMSOL multiphysics software to simulate the induced strain and piezoelectric potential on the surface of a strained BFO NS and NW (Figure S4). Based on these simulations, we observed that, under similar conditions a higher strain as well as a higher potential was generated on the surface of an NW than on an NS. This assumption was further confirmed by comparing the piezoelectric catalysis performance of BFO NWs with different lengths. With an increasing BFO NW length the corresponding *k*-values also increased (Figure S5). A similar trend was observed in the case of piezoelectric zinc oxide nanowires, whereby longer nanowires provided higher mechanical to chemical energy conversion efficiency for water splitting using ultrasound (US) (Hong et al., 2010). From Figure 4D, we further observe that when the BFO samples were excited by light and US simultaneously, the observed *k*-values (*k*_dual_) were higher in contrast to when light (*k*_light_) or US (*k*_us_) were solely used, and even higher than the linear addition of the two, especially for the BFO NWs. This increase in reaction rate offered by piezo-photocatalysis can be explained by an increase in the electron-hole pair formation activity because of a higher energy input available under the dual stimuli. The ability to degrade organic dyes (in the case of BFO NWs) can be greatly enhanced by a factor of 11.4 when a combination of light and mechanical energy is used simultaneously, with a *k*-value 0.0582 min^−1^ (Table S1 compares the reaction rate constants observed for different materials under light and ultrasonic vibrations). It is worth noting that under dual stimulation, the *k*-value (*k*_dual_) is slightly higher than the linear addition of the respective *k*-value under only light or US stimulation (*k*_light_ + *k*_us_), which indicates that the piezoelectric-driven internal field also helps the photogenerated carriers move to the surface of the BFO nanostructures.

Catalytic efficiency of BFO NWs to successfully harness the dual stimuli and degrade other organic dyes, i.e., methylene blue (MB), methyl orange (MO), and brilliant green (BG), at elevated dye concentrations of 10 mg L^−1^ was also investigated. Figure 4E presents a collage of images taken every 30 min to represent how the BFO NWs were able to degrade all four dyes within 2 hr. These results highlight the ability of BFO nanostructures to successfully scavenge energy from light and waste energy, such as noise or stray vibration, for water remediation (Figure S6). To develop photocatalysts for practical water purification applications, it is essential that they demonstrate not only good efficiency and versatility, but also high stability so that they can be recycled and reused. The reusability of BFO NS and NW samples was examined and is presented in Figure 4F. For both BFO samples we observe a slightly decreasing catalytic performance during the first three consecutive runs. It seems that the following two cycles did not further affect the performance. Long-term analysis will be carried out to examine how the catalytic stability of BFO changes over multiple cycles in future.

**Figure 5:**
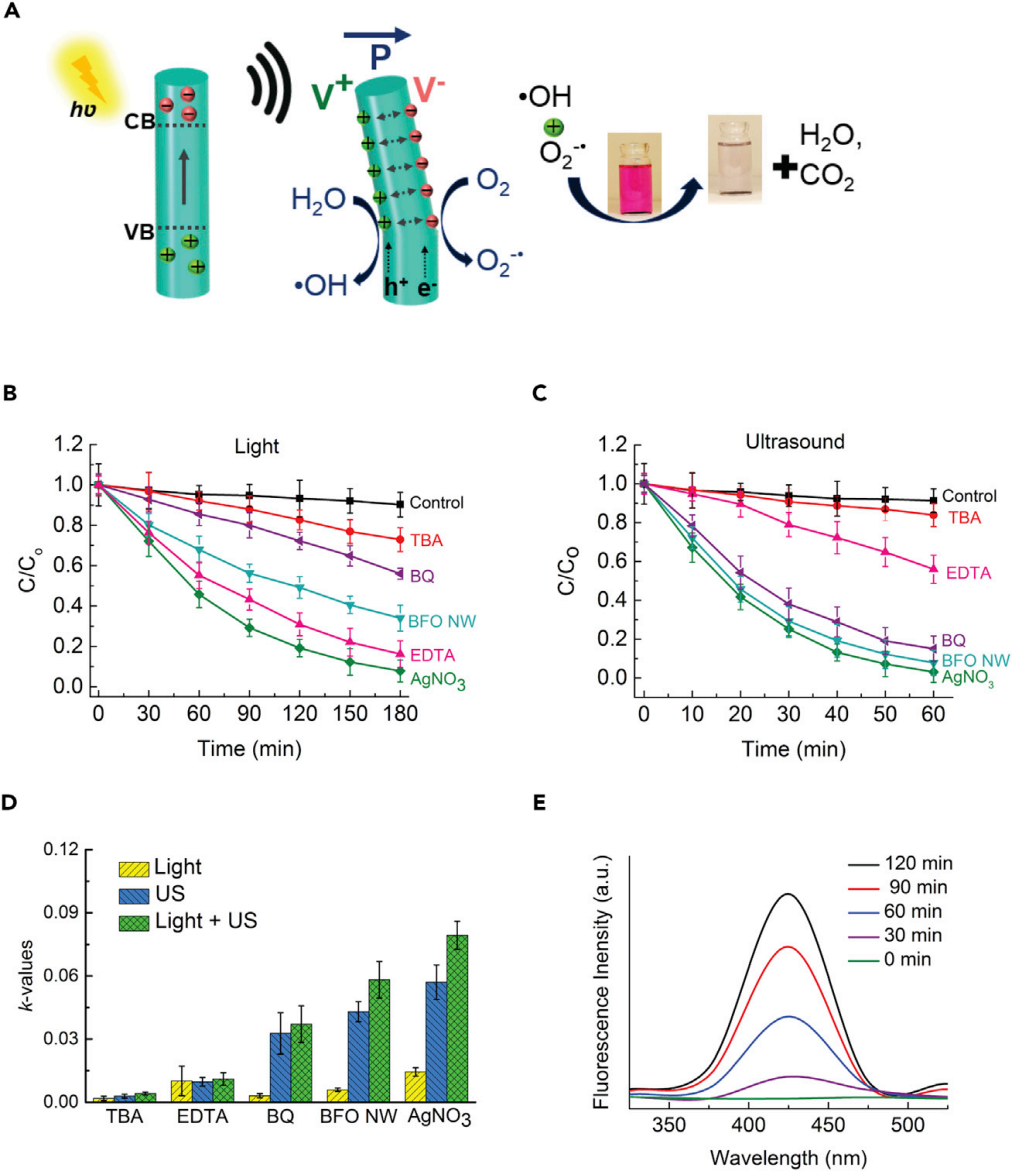
The Catalytic Degradation Mechanism of RhB by BFO (A) Catalytic degradation scheme of BFO NWs under light and ultrasound. (B) Trapping experiments performed to study catalytic degradation curves obtained for RhB under light (n = 5). (C) Trapping experiments performed under ultrasound (n = 5). (D) Comparison of degradation rate constants obtained for different scavengers under light, ultrasound, and their combination (n = 5). (E) Reaction of terephthalic acid with OH^⋅^ radicals to produce increasing amounts of fluorescent 2-hydroxyterephthalic acid with peak intensity at 425 nm.

To elucidate the possible RhB degradation pathway offered by BFO samples, we first investigated the interaction of the BFO surface with water. According to our DFT calculations, in the presence of a relaxed or strained BFO surface a water molecule can spontaneously dissociate into H^+^ and OH^−^ ions, followed by the highly favored adsorption of OH^−^ on the BFO surface (see Figure S7). The influence of an external stimulation (such as UV-visible light) leads to the formation of electron-hole pairs inside BFO samples (Figure 5A). Electrons are excited from the valence band (VB) of BFO to the conduction band (CB), leaving behind holes in the VB, and thus creating electron-hole pairs. These electron-hole pairs tend to recombine, leading to a decrease in the number of available charge carriers that can successfully migrate to the surface of BFO and initiate the redox reactions. When US is used to strain BFO, its internal piezoelectric field facilitates the separation of electric charge carriers and their migration toward the surface, thus increasing the probability of initiating the redox reactions. The various steps involved in the degradation of RhB dye by reactive species such as e^−^, h^+^, OH^⋅^, and O_2_^⋅-^ are the following:

(Equation 2) *BiFeO*_3_ + *hʋ* or US → *BiFeO*_3_ (*e*^−^ + *h*^+^)

(Equation 3) $$e^{-}+O_{2\;}\to O_{2}^{-\cdot }$$

(Equation 4) *h*^+^ + *H*_2_*O* → ⋅*OH*

(Equation 5) *h*^+^ + *RhB* → *degradation products*

(Equation 6) ⋅*OH* + *RhB* → *degradation products*

(Equation 7) $$O_{2}^{-\cdot }+RhB\;\to \;\mathrm{degradation}\;\mathrm{products}$$

To further determine how these reactive species were involved in the photocatalytic, piezocatalytic, and piezo-photocatalytic reactions, trapping experiments were performed. For this, degradation of RhB dye was carried out in the presence of BFO NWs and different scavengers of the reactive species (Table 1). It can be seen from Figure 5B that the photocatalytic degradation efficiency was increased with the addition of AgNO_3_ (an electron scavenger) and EDTA (a hole scavenger) (Zhang et al., 2016, Wu et al., 2016), which is probably caused by promoted separation of electron-hole pairs (and thus increased lifetime of electrons/holes) owing to their consumption, or caused by deposition of silver nanoparticles (Mohan et al., 2014). When benzoquinone (BQ) (a superoxide radical O_2_^⋅-^ scavenger) and tert-butyl alcohol (TBA) (a hydroxyl radical OH^⋅^ scavenger) were added, the photodegradation of RhB decreased. These results reveal that the predominant reactive species for photocatalytic degradation of RhB by BFO NW were the radicals. Trapping experiments were also performed to determine the main reactive species behind the piezocatalytic mechanism (Figure 5C). The addition of AgNO_3_ (electron scavenger) led to a slightly improved reaction rate, which is probably caused by reasons similar to the ones observed for the photocatalytic experiments (i.e., extended lifetime of holes or silver-nanoparticle-enhanced catalytic activity). However, when EDTA (hole scavenger) was added, a strong suppression of RhB degradation was observed, which indicates that holes play an important role in piezocatalytic degradation of RhB. Holes can either directly react with organic dyes to degrade them (Equation 5) or react with water to produce highly reactive OH^⋅^ radicals (Equation 4). Hence, their quenching can have a severe impact on RhB degradation. The severe suppression of RhB degradation rate by adding EDTA in the piezocatalytic experiment sharply contrasts with the increase in degradation activity when EDTA was introduced in the photocatalytic experiments. This result can be explained by the different concentration of charge carriers in these two experiments. In the piezocatalytic experiment, the charge carrier's concentration was much higher than that in the photocatalytic experiment, as evidenced by the higher degradation rate. When the holes were trapped, a severe suppression in RhB degradation rate overshadowed any potential degradation rate increase that could have been induced by the prolonged lifetime of electrons. Therefore, in the piezocatalytic experiments the degradation rate decreased compared with the pure BFO NW sample where no scavengers were introduced. However, it should be noted that, since the relative concentration (C/C_o_) of RhB after 1 hr in both experiments was approximately the same (i.e., about 60% RhB dye was still remaining), the degradation speeds for both photocatalytic and piezocatalytic experiments were the same. This result indicates that the degradation rate for both reactions was determined by the concentration of EDTA used for trapping the holes, which further corroborates our explanation. The trapping of OH^⋅^ radicals (that are produced by holes) shows similar trends in both catalytic experiments. The dominating role of holes can be further confirmed by the fact that O_2_^⋅-^ radicals, which are produced by electrons, exhibit a minimal impact on the piezocatalytic RhB degradation. The *k*-values obtained from the trapping experiments under stimulation of light, US, and the dual stimuli are presented in Figure 5D. For all degradation mechanisms, scavenging either holes or OH^⋅^ radicals led to a decrease in the reaction speed, whereas the electrons were the least active species for the degradation of RhB dye. In addition to the trapping experiments, we confirmed the formation of hydroxyl radicals in our degradation experiments by using terephthalic acid as a photoluminescent OH^⋅^-trapping agent. Terephthalic acid readily reacts with OH^⋅^ radicals to produce a highly fluorescent product, 2-hydroxyterephthalic acid, which emits a unique fluorescent signal at 425 nm (Wu et al., 2016). From the results of this experiment (Figure 5E) we can observe an increase in fluorescence intensity at 425 nm with increasing piezo-photocatalytic reaction time, which offers further proof of OH^⋅^ formation during the catalytic reaction.

**Table 1:** List of All the Scavengers Used in the Trapping Experiments and the Reactive Species They Quench

Scavenger	Reactive Species Quenched
AgNO_3_	e^-^
EDTA	h^+^
Tert-butyl alcohol (TBA)	OH^⋅^
Benzoquinone (BQ)	O_2_^⋅-^

## Conclusions

In this work, we have successfully fabricated single-crystalline BiFeO_3_ NSs and NWs that exhibit promising photocatalytic as well as piezocatalytic properties for water remediation. Both BFO nanostructures displayed a slow photocatalytic activity under UV-visible light, with the NS degrading RhB dye to 66% within 3 hr. Under piezocatalytic degradation of RhB, both BFO NSs and NWs were more efficient in degrading RhB, with an elevated efficiency of 59% and 91% within 1 hr, respectively. A comparison between the photocatalytic and piezocatalytic RhB degradation rates revealed that under ultrasonic vibrations, BFO NWs displayed an increase in degradation efficiency up to a factor of 8.5. When piezo-photocatalytic degradation was performed, the degradation efficiency of both BFO NS and NW samples was even higher, with *k*-values equal to 0.0209 and 0.0582 min^−1^, respectively. This increase in efficiency under piezoelectric stimulation may be caused by the internal piezoelectric-field-induced bias and its influence on suppressing the electron-hole recombination. Trapping experiments were performed to understand the degradation mechanism behind the photo- and piezocatalytic stimuli using scavengers for the reactive species. Holes and OH^⋅^ radicals were demonstrated to be the main reactive species. These results allow for the design of better catalysts that can readily and efficiently harness multiple sources of free energy, such as light and vibration, from their surroundings and use them for environmental applications.

## Methods

All methods can be found in the accompanying Transparent Methods supplemental file.

## Supplemental Information

Document S1. Transparent Methods, Figures S1–S9, and Table S1
