# Supplementary material for: Piezoelectrically Enhanced Photocatalysis with BiFeO3 Nanostructures for Efficient Water Remediation
Source: iScience. 2018 Jun 8;4:236–46. doi: 10.1016/j.isci.2018.06.003 (PMC6146592; doi:10.1016/j.isci.2018.06.003)
Supplement: Document S1. Transparent Methods, Figures S1–S9, and Table S1 [file mmc1.pdf]

ISCI, Volume *4*

Supplemental Information

Piezoelectrically Enhanced Photocatalysis with $BiFeO_3$ Nanostructures for Efficient Water Remediation

Fajer Mushtaq, Xiangzhong Chen, Marcus Hoop, Harun Torlakcik, Eva Pellicer, Jordi Sort, Chiara Gattinoni, Bradley J. Nelson, and Salvador Pané

Supplementary information

TRANSPARENT METHODS

Fabrication of $BiFeO_3$ nanostructures $BiFeO_3$ (BFO) nanosheets (NS) and nanowires (NW) were fabricated by a tunable hydrothermal synthesis approach. For the fabrication of BFO NS, $Bi(NO_3)\cdot 5H_2O$ and $FeCl_3\cdot 6H_2O$ were dissolved in a stoichiometric 1:1 molar ratio in acetone using continuous mechanical stirring and ultrasound. Next, DI water was added followed by concentrated ammonia under vigorous stirring to adjust the pH to 11. The resulting sediment was centrifuged, washed several times with DI water to reach a neutral pH value, and dissolved in 40 mL of DI water. NaOH (5 M) was added to the above solution and stirred for 1 h at room temperature. Finally, the above solution was transferred to a 50 mL sealed, Teflon-lined steel autoclave and heated at 140 ˚C for 72 h. The black powder obtained was washed with DI water and ethanol and dried at 60 ˚C for characterization.

For the fabrication of BFO NW, the same fabrication procedure was followed until the addition of 5 M NaOH. After this step, 5 mL of polyethylene glycol (Mn 400) was added to the 40 mL solution at 60 ˚C and stirred for 30 min. Finally, this solution was transferred to a 50 mL capacity sealed, Teflon-lined steel autoclaved and heated at 180 ˚C for 72 h. BFO NWs with different lengths were prepared by varying reaction times (details are provided in Figure S5).

Material characterization The morphology of the resulting BFO nanostructures was investigated by scanning electron microscopy (SEM, Zeiss ULTRA 55, Zeiss, Oberkochen, Germany), transmission electron microscopy (TEM, FEI F30, FEI Co., Hillsboro, OR), and scanning transmission electron microscopy (STEM, FEI F30). The crystallographic structure of the nanostructures was analyzed by X-ray diffraction on a Bruker AXS D8 Advance X-ray diffractometer, equipped with a Cu target with a wavelength of 1.542 Å. Cell parameters, crystallite sizes and microstrains were evaluated by Rietveld refinement using the Materials Analysis Using Diffraction (MAUD) software (overlapped experimental and computed patterns for both BFO NSs and NWS are provides in Figure S8).(Lutterotti et al., 1990) The local crystallographic structure was studied by selected area electron diffraction (SAED). Piezoresponse force microscopy (PFM) investigations were performed on a commercial atomic force microscope (NT-MDT Ntegra Prima). Pt-coated Si probes, i.e. HA-FM01/Pt, were used, and the imaging contact force set-points were carefully controlled. To perform PFM measurements on the long BFO NW, the nanowires were fixed by depositing carbon bands on their two ends using focused ion beam (FIB) (Figure S9). For domain imaging, AC signals (amplitude V_{AC} = 1 V) were used to excite the surface oscillations. To acquire local piezoresponse loops, ac signals (V_{AC} = 0.5 V) were superimposed on triangular staircase wave with DC switching from -10 V to 10 V. UV-Vis diffuse reflectance spectra (DRS) was acquired by a Cary 4000 UV-vis spectrophotometer. $BaSO_4$ was used as the reflectance standard.

Catalytic performance measurement Catalytic experiments were performed to study the degradation of RhB dye in the presence of our BFO structures. An RhB concentration of 4 mg L^{-1} was chosen to perform degradation experiments and 30 mg of BFO NSs and NWs were dispersed

in 30 mL of RhB solution under continuous agitation and the adsorption-desorption equilibrium was allowed to reach before starting the experiment. The reaction temperature was maintained at 25 ± 2 ˚C to negate the effect of temperature. For the photocatalytic experiments, the samples were irradiated with UV-visible light (300 nm $< \lambda <$ 600 nm) using a 300 W Xenon lamp (Asahi Spectra, MAX-303). For the piezo-catalytic measurements, samples were subjected to stress using an ultrasonic source (VWR USC300DF, nominal power and frequency: 80 W, 132 kHz) and kept in a dark environment (to avoid photocatalytic degradation). For the piezo-photocatalytic degradation experiments, the above mentioned light source was used in combination with the ultrasonic source. A UV-Vis spectrophotometer (Tecan Infinite 200 Pro) was used to obtain the fluorescent spectra of RhB over time by taking aliquots of irradiated RhB solution.

Trapping experiments To investigate the degradation pathways behind photocatalytic and piezocatalytic mechanism, we performed trapping experiments using different scavengers. $AgNO_3$ (2 mM), ethylene diamine tetraacetic acid (EDTA, 2 mM), tert-butyl alcohol (TBA, 2 mM) and benzoquinone (BQ, 0.5 mM) solutions were prepared in a 4 mg L^{-1} RhB solution. Next, experiments for photocatalysis, piezocatalysis and piezo-photocatalysis were performed in a similar manner as discussed above. For the experiment to probe the formation of $OH^{\bullet}$ radicals, 0.5 mM terephthalic acid solution was prepared in 30 mL DI water with 30 mg of BFO NWs and subjected to piezo-photocatalysis, after which the solution's intensity was monitored at 425 nm every 30 mins.

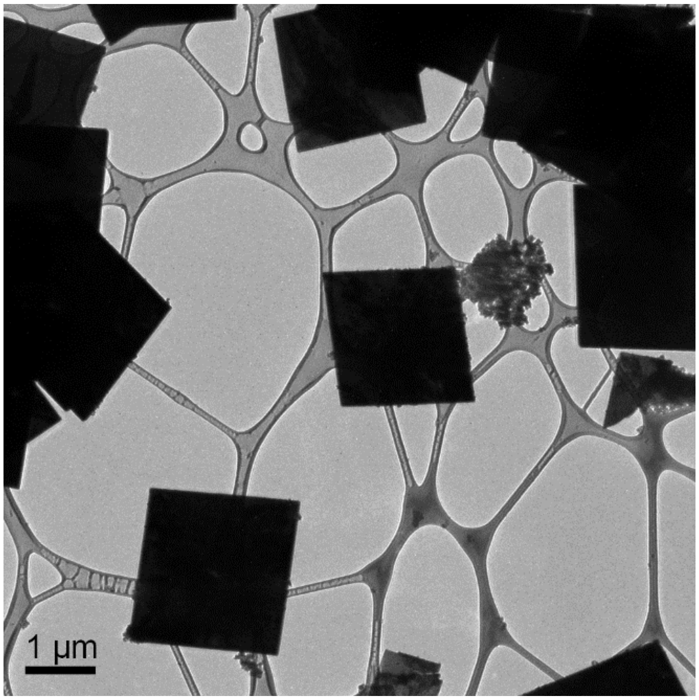

Figure S1: TEM image showing a few BFO NS, related to Figure 1.

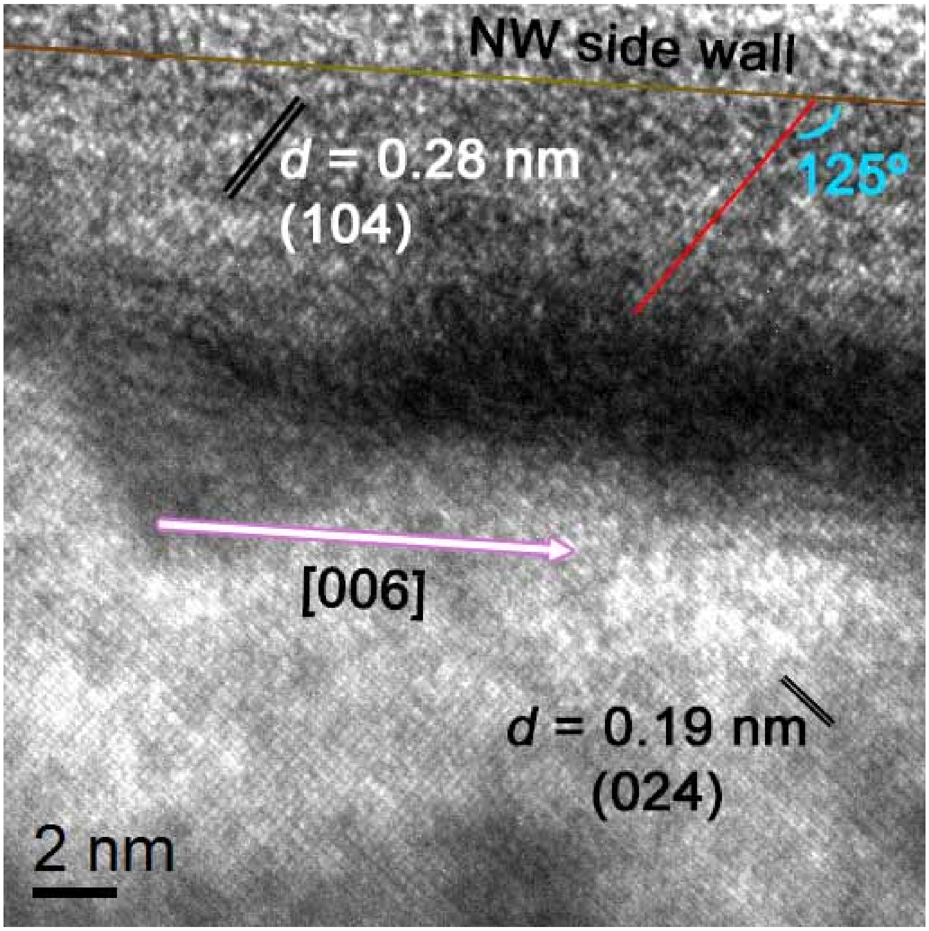

Figure S2: HRTEM image taken at the edge of a BFO NW showing its growth direction along the [006] axis, related to Figure 2.

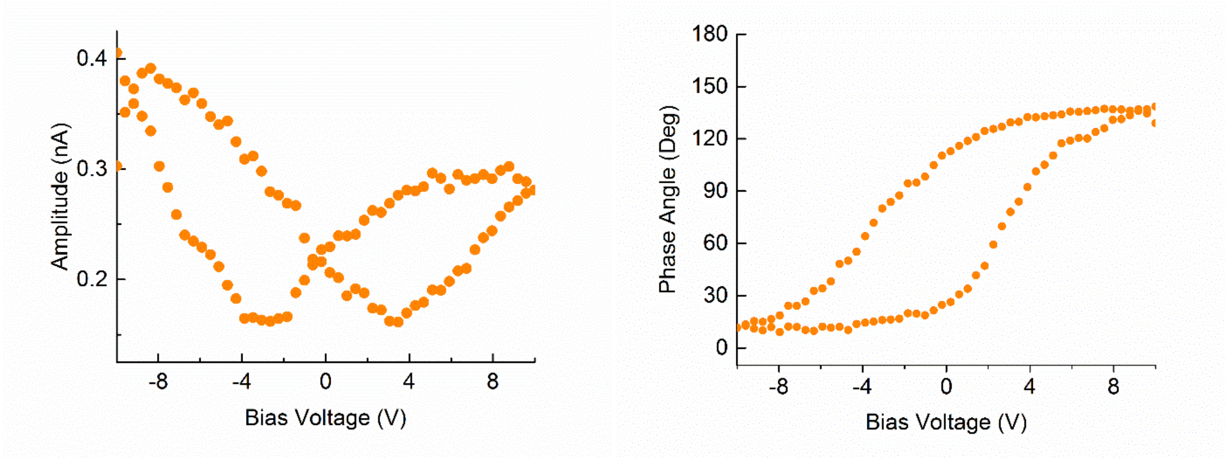

Figure S3: PFM analysis performed on a BFO NS, related to Figure 3.

Amplitude (left) and phase (right) loops obtained for a single BFO NS using PFM.

Multiphysics simulation and comparison of piezoelectric performance of BFO NS and NW

The theoretical piezo-electic performance of the BFO NS and NW in the degradation experiments was also investitaged in a multiphysics simulataion. The ultrasonic source (VWR USC300DF, nominal power and frequency: 80 W, 132 kHz) generates pressure waves with amplitudes up to 6.7 kPa calculated from reference. This pressure can induce compressive, tensile or shear stress in the multiferroic BFO structures.(Dai et al., 2017) The simulated BFO NS was implemented as a symmetric cuboid with a thickness of 145 nm and an edge length of 2.27 um, and BFO NW was implemented as a column with a thickness of 650 nm and a total length of 32 um (Young´s modulus of 189 GPa with a Poisson´s ratio of 0.35).(Aimon et al., 2012) The piezoelectric characteristics of this material with R3c symmetry is described by the piezo-electric coupling matrix

$$d = \begin{bmatrix} 13.5 & 0 & 0 & 0 & 9 & 0 \\ 0 & 13.5 & 0 & 9 & 0 & 0 \\ 3 & 3 & 50 & 0 & 0 & 0 \end{bmatrix} \cdot 10^{-12} [\mathrm{C/N}]$$

and the compliance matrix

$$s_E = \begin{bmatrix} 0.0182167 & -0.0006753 & -0.0179691 & 0.0011707 & 0 & 0 \\ \cdot & 0.0182167 & -0.0179691 & -0.0011707 & 0 & 0 \\ \cdot & \cdot & 0.0492076 & 0 & 0 & 0 \\ \cdot & \cdot & \cdot & 0.0192123 & 0 & 0 \\ \cdot & \cdot & \cdot & \cdot & 0.0192123 & 2 \cdot 0.0011707 \\ \cdot & \cdot & \cdot & \cdot & \cdot & 0.0377839 \end{bmatrix} \cdot 10^{-9}[1/Pa]$$

which were determined from literature.(Graf et al., 2015, Nye, 2009, Aimon et al., 2012, Wang et al., 2009) As the material is not isotropic, the preferred growth directions of [024] for the BFO NS and [006] for the BFO NW had to be taken into account. The mechanical and electrical boundary conditions were set on the back face of the BFO NS and the bottom face of the BFO NW. Since the geometric shape of both BFO structures have several symmetries, multiple ways of applying stress on to the suface exist such as the compressive, tensile and shear. Here, we present the most pronounced combinations for each structure. On the basis of the results obtained, we can conclude that for both BFO NS and BFO NW, an application of a shear stress results in the highest generated surface potential, where a force per unit area of 6.7 kPa is simultaneously applied on the top surface of the structures along the positive x-axis and on the front left surface along the negative z-axis (Figure S4 f,l). In every scenario, BFO NW expereinces higher induced potentials than the NS sample. This suggests that the shape of the BFO structure can influnce its piezo-electric performance, which is also supported by the results obtained from the degradation experiments.

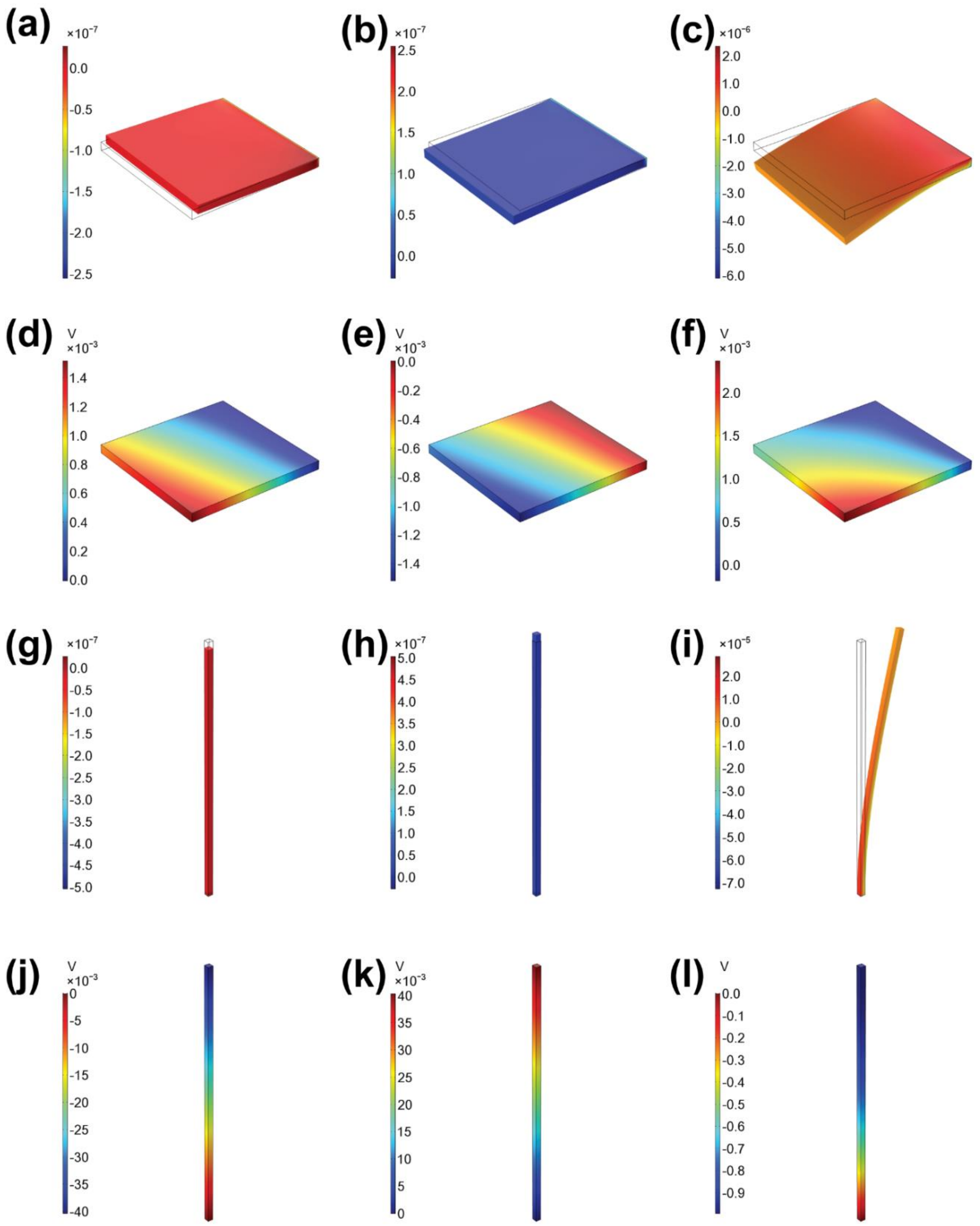

Figure S4: COMSOL multiphysics simulations obtained for BFO NS and NW showing different deformations, related to Figure 4.

(a) under compressive, (b) tensile and (c) shear stress with the correspondingly induced piezoelectric potential (d-f). Similarly, for BFO NW, the strain under (g) compressive, (h) tensile, and (i) shear stress and the correspondingly induced piezoelectric potential (j-l) are also presented.

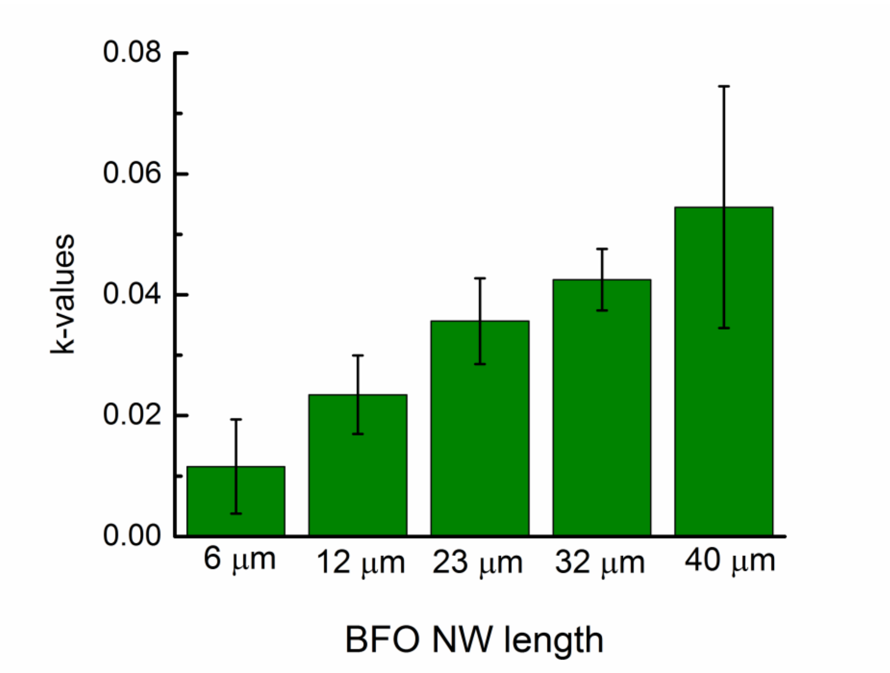

Figure S5: RhB degradation rate constant values obtained under piezo-catalysis for BFO NW with increasing lengths, related to Figure 4.

The different lengths were prepared by varying reaction times i.e. 12 hr (6 µm), 24 hr (12 µm), 48 hr (23 µm), 72 hr (32 µm) and 80 hr (40 µm).

Architecture/Composition	Energy source	k-value (min^{-1})	Ref.
Coaxial TiO_2-PtPd NWs	Light: 450 W, Xe lamp<br>λ > 420 nm	0.071	(Mushtaq et al., 2016)
Metal- TiO_2 core-shell NPs	Light: 300 W, Xe lamp<br>λ > 400 nm	0.013 (TiO_2-Au)<br>0.038 (TiO_2-Pd)<br>0.016 (TiO_2-Pt)	(Zhang et al., 2011)
Polyaniline (PANI)/TiO_2 nanocomposite	Light: 100 W, W-halogen lamp, λ > 400 nm	0.052 (PANI-TiO_2)<br>0.0035 (TiO_2)	(Masid et al., 2015)
Nanopowders of novel photocatalysts	Light: 500 W, Xe lamp 400 nm < λ < 800 nm	0.019 ($ZnBiSbO_4$)<br>0.004 (N-TiO_2)<br>0.005 ($CdBiYO_4$)	(Luan et al., 2014)
MWCNT/TiO_2 composite	Light: 500 W, halogen lamp, λ > 400 nm	0.0036 (TiO_2)<br>0.0149 (MWCNT-TiO_2)	(Abdullahi et al., 2015)
TiO_2/CuS Core/Shell nanowires	Light: 500 W, Xe lamp<br>λ > 420 nm	0.0019 (TiO_2)<br>0.014 (CuS NPs on TiO_2)<br>0.036 (Core-shell TiO_2-CuS)	(Khanchandani et al., 2016)
FeS/ZnO nanowire array	Light: 500 W, Xe lamp<br>US: 200 W	2.5 (100 mg loading)	(Xiao et al., 2016)
CuS/ZnO nanowire array	Light: 500 W, Xe lamp<br>200 nm < λ < 1100 nm<br>US: 200 W	4 (100 mg loading)	(Hong et al., 2016)
Single-phase $BiFeO_3$ nanostructures	Light: 450 W, Xe lamp<br>US: 80 W	0.06	This work

Table S1 Comparison of rate constants obtained for various catalysts under visible light and ultrasonic vibrations. Related to Figure 4

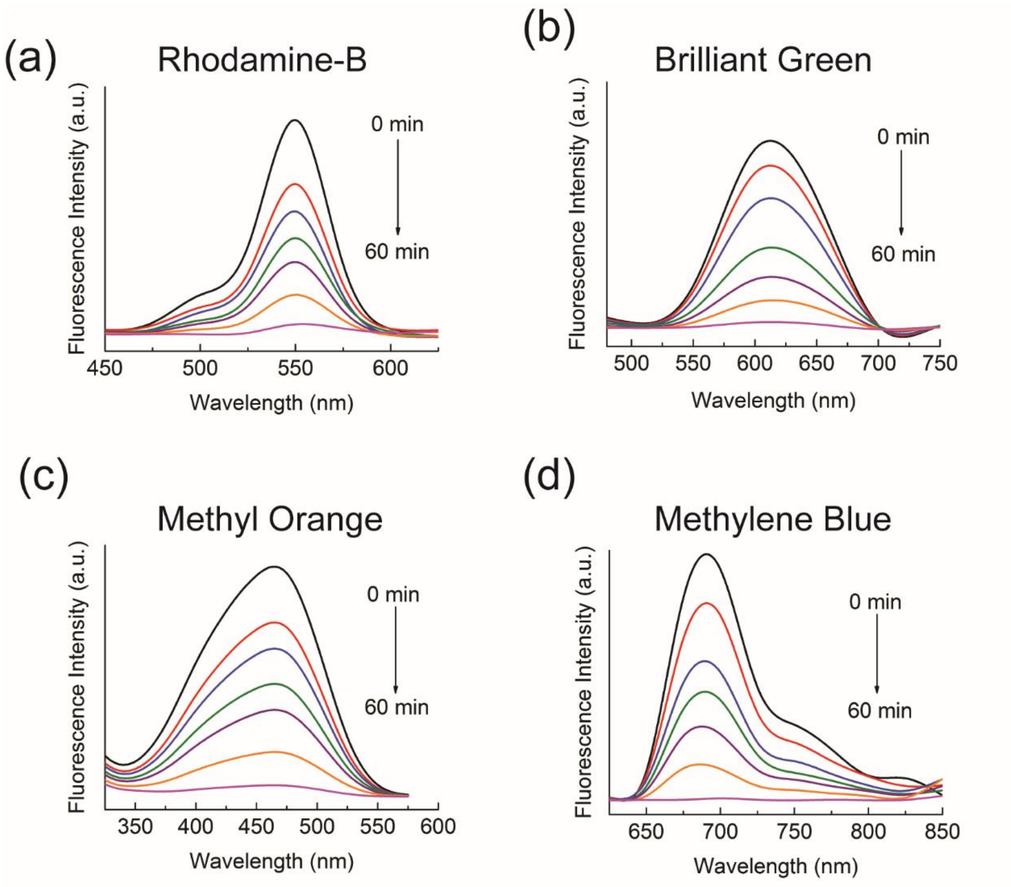

Figure S6: Piezo-photocatalytic degradation of four different organic dyes using BFO NWs under stimulation for 1 h, related to Figure 4.

DFT calculation on water adsorption on BiO-terminated (100) surface

Density functional theory (DFT) calculations were performed with VASP.(Kresse et al., 1993, Kresse et al., 1996a, Kresse et al., 1996b) The inner core electrons were replaced by projected atomic waves(Kresse et al., 1999) and the optB86b-vdW functional(Jiří et al., 2010) was used. OptB86b-vdW is a non-local functional which accounts for long-range van der Waals interaction and it is part of the vdW-DF family of functionals(Dion et al., 2004) which have been shown to perform well for the adsorption of water and other small molecules, especially on metals(Klimeš et al., 2012, Gattinoni et al., 2015, Liriano et al., 2017, Carrasco et al., 2011, Carrasco et al., 2014). A plane wave cutoff of 500eV and a 5x5x5 Mokhorst-Pack k-points mesh was used. On-site Coulomb interaction in the Fe d orbitals were taken into account by the addition of a Hubbard U, in the Dudarev approach(Liriano et al., 2017) with a U-J=4 eV. This setup led to a good description of bulk BFO with the lattice constant a, and rhombohedral angle α being within 1% of the experimental values (measured values for a and α are 3.975 and 59.59, while the experimental

values are 3.983 and 59.35, respectively). The magnetic moment, μ values were within 10% of the experimental values, with the measured value being 4.10 and the experimental value being 3.75. The value of the band gap, 2.25 eV, is within the experimentally measured range (1.3-3.0)(Higuchi et al., 2008, Reda et al., 2012) and close to the band gap in the nanocrystals presented in this paper.

A slab geometry with a (001) surface normal and a 2x2 area is employed. We introduce ~15 Å of vacuum between periodic images of the slab in the z direction. The k-points mesh in the slabs is 2x2x1. The convergence criterion on the forces is 5x 10^{-3} eV/ Å. The bottom to layers of the slab are fixed to bulk position while the atoms in the upmost layers are allowed to relax. Seven-layer-thick slabs were sufficient to converge the cleavage energy ($E_{cl}=E_{BiO}-E_{FeO2}-nxE_{bulk}$) within 10 meV and were thus employed in all calculations. The adsorption energies were calculated as: $E_{ads}=E_{whole}-E_{slab}-E_{H2O}$, where E_{whole}, E_{slab} and E_{H2O} are the total energies of, respectively, the whole adsorbed system, the substrate and the water molecule in the gas phase.

Two surface terminations are possible, a BiO and a FeO_2 termination. We have focused on the BiO-terminated surface. Two polarization direction are possible, up and down. A drive for the dissociation of water was defined as a more negative adsorption energy of a co-adsorbed OH and H rather than for an intact H_2O.

A propensity for dissociation was observed for the up-polarized surface, where the adsorption energy for the intact molecule is E_{ads}=-2.98 eV and for the dissociated one is E_{ads}=-3.03 eV. This holds true for a range of strains of the substrate, between -5% and +5%.

In the case of the down-polarized surface, a drive for dissociation was not observed, the adsorption energy of the intact molecule being E_{ads}=-3.01 eV and for the dissociated molecule E_{ads}=-2.36 eV.

In all cases the most stable adsorption configuration was found to be, for the intact (dissociated) structure, with the H_2O (OH) sitting in the bridge position between two Bi atoms with on H atom pointing towards (adsorbed to) the neighboring O atom (as shown in Fig. S7).

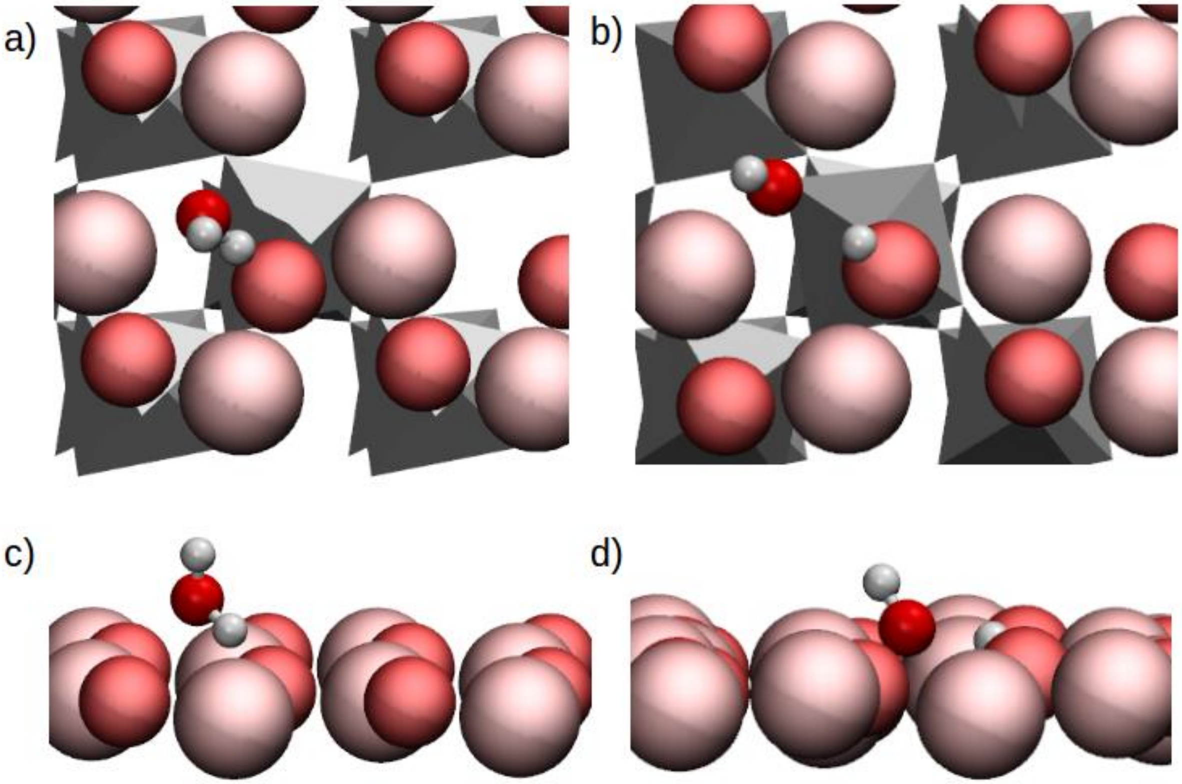

Figure S7: Adsorption structure of intact and dissociated water on the BiO-terminated (100) BFO surface. Both up and down polarized surfaces present the same adsorption structure, related to Figure 5.

(a) Top and (c) side view of a single H_2O adsorbed in the Bi-Bi bridge position.

(b) Top and (d) side view of a dissociated HO+H. O is in red, H in white and Bi in pink. Only the top layer atoms of BFO are shown, the other layers are only shown with the grey oxygen octahedral.

(a)

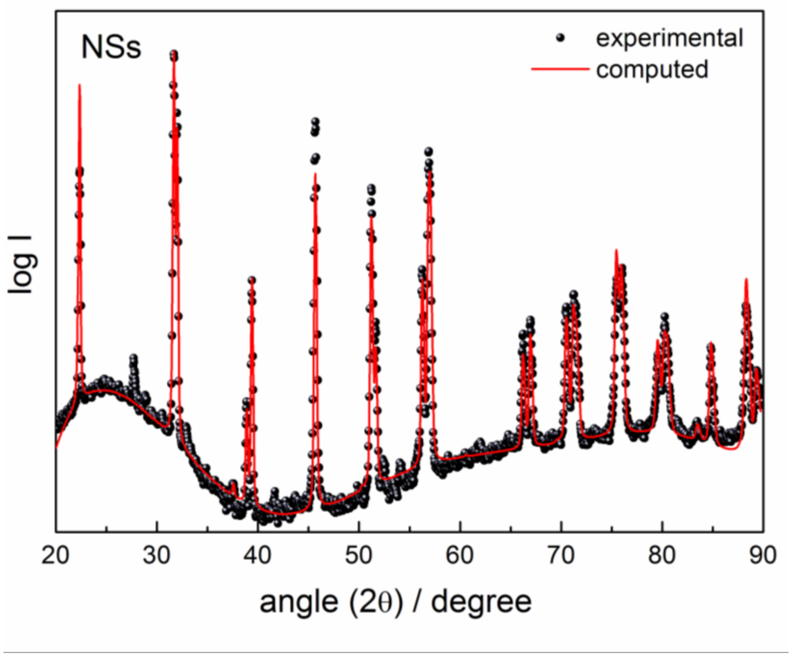

(b)

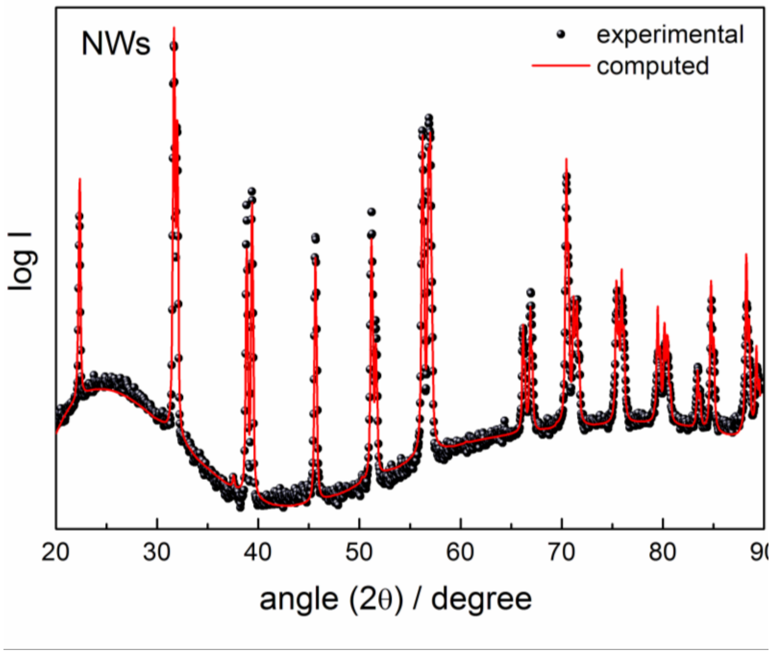

Figure S8: Rietveld analysis performed by MAUD software with overlapped experimental and computed XRD pattern, related to Figure 2.

(a) BFO NSs. (b) BFO NWs.

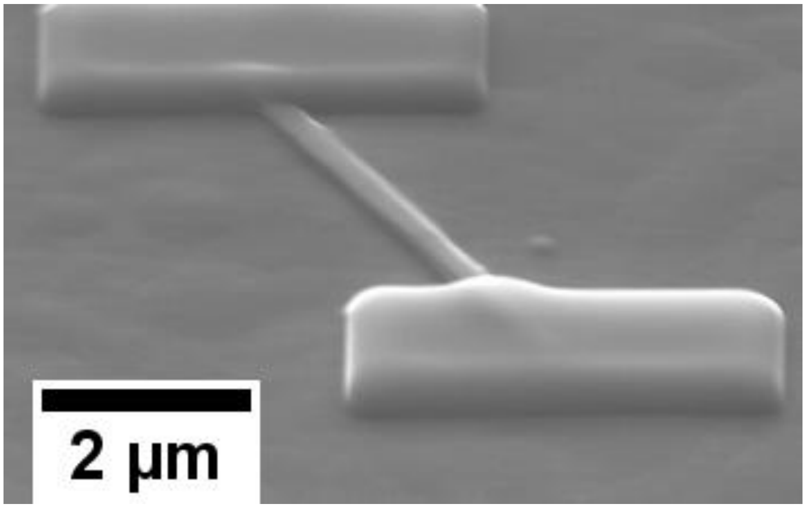

Figure S9: An SEM image showing a BFO NW fixed by carbon bands deposited using FIB, related to Figure 3.

SUPPLEMENTAL REFERENCES

Abdullahi, N., Saion, E., Shaari, A. H., Al-Hada, N. M. and Keiteb, A. (2015). Optimisation of the Photonic Efficiency of TiO_2 Decorated on MWCNTs for Methylene Blue Photodegradation. PLoS ONE 10, 125511.

Aimon, N. M., Liao, J. and Ross, C. A. (2012). Simulation of inhomogeneous magnetoelastic anisotropy in ferroelectric/ferromagnetic nanocomposites. App. Phys. Lett. 101, 232901.

Carrasco, J., Liu, W., Michaelides, A. and Tkatchenko, A. (2014). Insight into the description of van der Waals forces for benzene adsorption on transition metal (111) surfaces. J. Chem. Phys. 140, 084704.

Carrasco, J., Santra, B., Klimeš, J. and Michaelides, A. (2011). To Wet or Not to Wet? Dispersion Forces Tip the Balance for Water Ice on Metals. Phys. Rev. Lett. 106, 026101.

Dai, B., Lu, C., Kou, J., Xu, Z. and Wang, F. (2017). Photocatalytic performance of PMN-PT@TiO_2 highly enhanced by alternative spatial electric field induced charge separation effect. J. Alloys Compd. 696, 988-995.

Dion, M., Rydberg, H., Schröder, E., Langreth, D. C. and Lundqvist, B. I. (2004). Van der Waals Density Functional for General Geometries. Phy. Rev. Lett. 92, 246401.

Gattinoni, C. and Michaelides, A. (2015). Understanding corrosion inhibition with van der Waals DFT methods: the case of benzotriazole. Faraday Discuss. 180, 439-458.

Graf, M., Sepliarsky, M., Machado, R. and Stachiotti, M. G. (2015). Dielectric and piezoelectric properties of $BiFeO_3$ from molecular dynamics simulations. Solid State Commun. 218, 10-13.

Higuchi, T., Liu, Y.-S., Yao, P., Glans, P.-A., Guo, J., Chang, C., Wu, Z., Sakamoto, W., Itoh, N., Shimura, T., Yogo, T. and Hattori, T. (2008). Electronic structure of multiferroic $BiFeO_3$ by resonant soft x-ray emission spectroscopy. Phys. Rev. B 78, 085106.

Hong, D., Zang, W., Guo, X., Fu, Y., He, H., Sun, J., Xing, L., Liu, B. and Xue, X. (2016). High Piezo-photocatalytic Efficiency of CuS/ZnO Nanowires Using Both Solar and Mechanical Energy for Degrading Organic Dye. ACS Appl. Mater. Inter. 8, 21302-21314.

Jiří, K., David, R. B. and Angelos, M. (2010). Chemical accuracy for the van der Waals density functional. J. Phys. Condens. Matter. 22, 022201.

Khanchandani, S., Kumar, S. and Ganguli, A. K. (2016). Comparative Study of TiO_2/CuS Core/Shell and Composite Nanostructures for Efficient Visible Light Photocatalysis. ACS Sustain. Chem. Eng. 4, 1487-1499.

Klimeš, J. and Michaelides, A. (2012). Perspective: Advances and challenges in treating van der Waals dispersion forces in density functional theory. J. Chem. Phys. 137, 120901.

Kresse, G. and Furthmüller, J. (1996a). Efficiency of ab-initio total energy calculations for metals and semiconductors using a plane-wave basis set. Comput. Mater. Sci. 6, 15-50.

Kresse, G. and Furthmüller, J. (1996b). Efficient iterative schemes for ab initio total-energy calculations using a plane-wave basis set. Phys. Rev. B 54, 11169-11186.

Kresse, G. and Hafner, J. (1993). Ab initio molecular dynamics for liquid metals. Phys. Rev. B 47, 558-561.

Kresse, G. and Joubert, D. (1999). From ultrasoft pseudopotentials to the projector augmented-wave method. Phys. Rev. B 59, 1758-1775.

Liriano, M. L., Gattinoni, C., Lewis, E. A., Murphy, C. J., Sykes, E. C. H. and Michaelides, A. (2017). Water–Ice Analogues of Polycyclic Aromatic Hydrocarbons: Water Nanoclusters on Cu(111). J. Am. Chem. Soc. 139, 6403-6410.

Luan, J., Chen, M. and Hu, W. (2014). Synthesis, Characterization and Photocatalytic Activity of New Photocatalyst $ZnBiSbO_4$ under Visible Light Irradiation. Int. J. Mol. Sci. 15, 9459-9480.

Lutterotti, L. and Scardi, P. (1990). Simultaneous structure and size–strain refinement by the Rietveld method. J. App. Cryst. 23, 246-252.

Masid, S., Tayade, R. and Rao, N. N. (2015). Efficient visible light active Polyaniline/TiO_2 nanocomposite photocatalyst for degradation of Reactive Blue 4. Inter. J. Photocatal. Photon 119, 190-203.

Mushtaq, F., Asani, A., Hoop, M., Chen, X.-Z., Ahmed, D., Nelson, B. J. and Pané, S. (2016). Highly Efficient Coaxial TiO_2-PtPd Tubular Nanomachines for Photocatalytic Water Purification with Multiple Locomotion Strategies. Adv. Funct. Mater. 26, 6995-7002.

Nye, J. F. 2009. *Physical properties of crystals : their representation by tensors and matrices*, Oxford : Clarendon Press.

Reda, M., Guy, S., Olivier, R., Dorothée, C. and Michel, V. (2012). Photoluminescence Investigation of Defects and Optical Band Gap in Multiferroic $BiFeO_3$ Single Crystals. Appl. Phys. Express 5, 035802.

Wang, Y. L., Wu, Z. H., Deng, Z. C., Chu, L. Z., Liu, B. T., Liang, W. H. and Fu, G. S. (2009). First-Principle Calculation of Elastic Compliance Coefficients for $BiFeO_3$. Ferroelectrics 386, 133-138.

Xiao, G., Yongming, F., Deyi, H., Binwei, Y., Haoxuan, H., Qiang, W., Lili, X. and Xinyu, X. (2016). High-efficiency sono-solar-induced degradation of organic dye by the piezophototronic/photocatalytic coupling effect of FeS/ZnO nanoarrays. Nanotechnology 27, 375704.

Zhang, N., Liu, S., Fu, X. and Xu, Y.-J. (2011). Synthesis of M@TiO_2 (M = Au, Pd, Pt) Core–Shell Nanocomposites with Tunable Photoreactivity. J. Phys. Chem. C 115, 9136-9145.
